# Development and validation of a shortened version of the Child Abuse Self Report Scale (CASRS-12) in the Arabic language

**DOI:** 10.1186/s13034-022-00533-3

**Published:** 2022-12-09

**Authors:** Feten Fekih-Romdhane, Mariam Dabbous, Rabih Hallit, Diana Malaeb, Toni Sawma, Sahar Obeid, Souheil Hallit

**Affiliations:** 1 The Tunisian Center of Early Intervention in Psychosis, Department of Psychiatry “Ibn Omrane”, Razi Hospital, 2010 Manouba, Tunisia; 2 Faculty of Medicine of Tunis, Tunis El Manar University, Tunis, Tunisia; 3 School of Pharmacy, Lebanese International University, Beirut, Lebanon; 4 School of Medicine and Medical Sciences, Holy Spirit University of Kaslik, P.O. Box 446, Jounieh, Lebanon; 5 Department of Infectious Disease, Bellevue Medical Center, Mansourieh, Lebanon; 6 Department of Infectious Disease, Notre Dame des Secours University Hospital, Byblos, Postal Code 3 Lebanon; 7 College of Pharmacy, Gulf Medical University, Ajman, United Arab Emirates; 8 School of Arts and Sciences, Social and Education Sciences Department, Lebanese American University, Jbeil, Lebanon; 9 Research Department, Psychiatric Hospital of the Cross, Jal Eddib, Lebanon; 10 Applied Science Research Center, Applied Science Private University, Amman, Jordan; 11 Psychology Department, College of Humanities, Effat University, Jeddah, 21478 Saudi Arabia

**Keywords:** Child abuse, Child Abuse Self Report Scale, CASRS, Short form, Arabic, Psychometric properties

## Abstract

**Background:**

All available scales to screen for child abuse may be challenging to administer due to their length. Indeed, a higher number of items is associated with more administration time and less motivation to engage in responding. We aimed through this study to examine the psychometric properties of a brief Arabic version of the Child Abuse Self Report Scale (CASRS-12) in terms of factorial structure, internal consistency, divergent validity, and correlations with measures of bullying victimization, eating attitudes and perceived social support.

**Methods:**

We performed a cross-sectional, web-based study among Community Lebanese adolescents; where two samples have been used (Sample 1: N = 852, aged 15.34 ± 1.18 years, 54.8% females; Sample 2: N = 404, aged 16.60 ± 1.51 years, 57.2% females). The construct validity was tested using both exploratory and confirmatory factor analyses.

**Results:**

Results revealed that both EFA and CFA yielded a four-factor structure for the CASRS-12 that mirrors the original four factors captured by the original CASRS. The scale also showed a good internal consistency as evidenced through McDonald’s ω values ranging from 0.87 to 0.93 for the four subscales; and configural, metric, and scalar invariance across gender. Finally, we found that all CASRS-12 dimensions were significantly and positively correlated with more inappropriate eating attitudes and lower social support; and that psychological, physical, and sexual abuse correlated with higher bullying victimization

**Conclusion:**

In light of these findings, we potentially encourage clinicians and researchers to use this scale as a valid and reliable measure of child abuse among Arabic-speaking populations.

## Introduction

Child maltreatment refers to “all forms of physical and emotional ill-treatment, sexual abuse, neglect, and exploitation that results in actual or potential harm to the child’s health, development or dignity” [1, 2]. Four main types of child abuse can be identified: physical, sexual, and psychological abuse (acts of commission), and neglect (act of omission in the care) [3]. A systematic review and meta-analysis estimated that the global number of children aged between 2 and 17 years old who were victims of any form of abuse (physical, emotional and sexual) was of over 1 billion [3]. There is a strong evidence that child abuse contributes to short- and long-term detrimental consequences on physical and mental health [4–7]. Beyond the immediate pain and hurt it engenders, child abuse causes a wide range of negative effects, including bodily pain poor general health, depression, anxiety [8], suicide ideation [9], posttraumatic stress symptoms, dissociation, aggression, social withdrawal, school absences, suspension, or withdrawal [10], life-lasting cognitive deficits [11], bullying victimization [12, 13], violence perpetration and crime [14]. Child abuse was referred to as the “invisible epidemic” [15], since it has affected and continues to affect a substantial proportion of children worldwide [16]. As such, child abuse has been identified by the World Health Organization as a major risk factor related to the global burden of disease [17]. It has also been recognized since decades and globally as major, but preventable, public health and human rights problem [18]. The problem is more critical in some contexts such as low- and middle-income countries [19], more particularly Arab countries [20].

Arab countries are demographically one of the most youthful countries in the world [21]. Physical and psychological violence against children as a form of discipline is largely normalized and accepted in Arab cultures, and is not legally prohibited in the vast majority of Arab countries [22]. Due to the many conflicts in the region, the new Arab generations have known a dramatic increase in collective violence during the last years [22]. The UNICEF estimated that, in 2015, more than 70% of the world’s adolescents who died due to collective violence live in the Middle East and North Africa, and 7 in 10 children living in the region are physically abused [22]. Therefore, efforts to effectively assess, monitor, and manage the consequences of child abuse and neglect on Arab adolescents and young adults should be prioritized in the region. In addition, and given the magnitude of the problem, academic institutions have been called upon to increase research capacity to build a local evidence-base on violence against children in Arab countries that helps inform policies and interventions [23]. The first step to this end is to provide valid measurement instruments that can be used in Arab settings to evaluate the multidimensional and complex construct of child abuse.

A few scales have been previously used in Arab studies, including the 30-item Adverse Childhood Experiences-International Questionnaire (ACE-IQ) [24] (e.g., in Saudi Arabia [25], Iraq [26], Tunisia [27]), the 28-item Childhood Trauma Questionnaire (CTQ) [28] (e.g., in Tunisia [29, 30], Saudi Arabia [31], Egypt [32]), and the Child Abuse Self Report Scale (CASRS) [33] (e.g., in Lebanon [34]). The CASRS consists of 38 items that load onto four subscales assessing four abuse dimensions: (1) Physical abuse (physical punishment/abuse), (2) Psychological abuse (acts leading to fear or psychological pain), Sexual abuse (unwanted sexual touch and forced sexual contact by an adult or older child, including family members), and Neglect (lack of engaging in behaviours that are necessary to meet the developmental needs of a child, e.g. failure to provide adequate supervision or food) [33]. All these dimensions are accounted for, regardless of whether there was injury or damage caused [33]. The CASRS has previously been translated by our team using the forward and backward method [34], and has exhibited excellent psychometric properties in both Arabic-speaking clinical [35] and non-clinical populations [34, 36]. However, in the specific context of abuse and trauma, all these scales may be challenging to administer due to their length. There have been a very few validation studies of short child maltreatment measures (e.g., [37, 38]); however, no brief forms exist in Arabic so far. A higher number of items is associated with more administration time and less motivation to engage in responding. Longer scales are thus linked to lower quality responses, higher refusals and drop-outs, and lower response rates lower [39]; in addition to being costly. One strategy to overcome these disadvantages is to develop briefer forms that offer the potential benefits of being more practical, easier to interpret, less time-consuming and burdening, less costly; while trying at the same time to preserve the same measurement precision and standards of psychometric excellence of the full-length version [40]. In this perspective, we sought to develop a brief version of the Arabic CASRS that would assess the construct of child abuse in an appropriate and valid way. We thus aimed to explore its factorial structure, internal consistency, divergent validity, and correlations with other measures. We hypothesized that the Arabic CASRS-12 would (1) reproduce the four-factor structure identified by the developers of the original 38-item CASRS, (2) show adequate validity and reliability, (3) be invariant across gender.

## Methods

### Participants

A total of 841 Lebanese adolescents enrolled in Study 1, whereas 404 adolescents enrolled in Study 2. The description of the sociodemographic variables and other covariates for both samples are summarized in Table 1.

**Table 1:** Description of the two samples

Variable	Sample 1 (N = 841)	Sample 2 (N = 404)
Female sex (N, %)	443 (52.7%)	231 (57.2%)
Age, years (Mean ± SD)	15.41 ± 1.06	16.60 ± 1.51
Psychological abuse	2.33 ± 2.67	1.54 ± 2.16
Neglect	3.69 ± 2.97	3.81 ± 2.95
Physical abuse	1.95 ± 2.55	1.38 ± 2.19
Sexual abuse	1.62 ± 2.36	1.35 ± 2.21
Bullying victimization	−	3.64 ± 5.06
Social support	−	57.03 ± 19.87
Eating attitudes	−	17.58 ± 18.17

### Measures

#### Child Abuse Self Report Scale

The CASRS-12 items in English are reported in Table 1 and the items in Arabic are reported in Appendix 1. Regarding the scale shortening procedure, the initial pool of 38 items of the original scale was reviewed for relevance by an expert panel. The expert panel was composed of specialist clinicians in the field of childhood trauma from both countries (Lebanon and Tunisia), including authors FFR, SO and SH. The expert review included two main aspects: (1) relevance of items to each dimension of the scale, and (2) suitability for the Arab cultural context. Items that were similar in wording with another item were excluded to reduce redundancy (i.e. “I am beaten up because of every small mistake” and “When my parents punish me, it is not proportionate to my mistakes”). Items that correlated highly (r > 0.90) with another item were also excluded. Inter-item correlations and Item-total correlations were obtained from the study using the Arabic 38-item CASRS [41] and from Study 1 of this project. An item preselection was complemented by a factor analysis on the remaining pool of 12 items. As such, predictive validity and replication of the four-factor structure of the scale were also considered in selecting items.

#### Illinois bullying victimization

Permission to use the scale was obtained from Dr Dorothy Espelage. Validated in Lebanon [41, 42], it consists of sixteen items yielding two subscales, bullying perpetration (e.g. “I annoyed other students”) and bullying victimization (e.g. “Other students beat and pushed me”) [43]. Questions are rated from 0 = never to 4 = up to seven times or more. Higher scores reflect higher bullying perpetration and victimization respectively [44]. In this study, we used the victimization bullying subscale only (ω = 0.92).

#### Multidimensional social support scale

It is a succinct research instrument, gauging the degree of individual perceptions of social support that emanates from three distinct sources: Family, Friends and a Significant Other—measured by three subscales of four items each. Items include “My family really tries to help me”, “I can count on my friends when things go wrong” and “There is a special person in my life who cares about my feelings”. Higher scores express stronger feelings of being socially supported [45]. This scale has also been validated among Lebanese adults [46, 47] (ω = 0.98).

##### Disordered eating

Participants were asked to complete the Eating Attitudes Test-7, a 7-item measure of symptoms and concerns characteristic of eating disorders [48]. All items were rated on a 6-point scale, ranging from 1 (never) to 6 (always). Higher total scores reflect greater disordered eating attitudes (ω = 0.91).

##### Demographics

Participants were asked to provide their demographic details consisting of age and sex.

### Procedures

Ethics approval for this study was obtained from the Psychiatric Hospital of the Cross ethics committee (Approval Code: HPC-024–2022). Written informed consent was obtained from all subjects and or their legal guardians for study participation; the online submission of the soft copy was considered equivalent to receiving a written informed consent. All data were collected via a Google Form link, between July and August 2022. The project was advertised on social media (WhatsApp and Facebook) and included an estimated duration. The researchers approached adolescents they know directly; participants were asked to share the link with other adolescents they might know (friends and family members). This procedure was followed for the two samples. Inclusion criteria for participation included being Lebanese of origin and aged between 12 and 18 years. Internet protocol (IP) addresses were examined to ensure that no participant took the survey more than once. After providing digital informed consent, participants were asked to complete the instruments described above, which were presented in a pre-randomised order to control for order effects. The survey was anonymous and participants completed the survey voluntarily and without remuneration.

### Analytic strategy

#### Data treatment

There were no missing responses in the dataset. To examine the factor structure of the CASRS-12, we used an EFA-to-CFA strategy [49]. Sample 1 was used for the EFA and Sample 2 for the CFA.

#### Exploratory factor analysis

To explore the factor structure of CASRS-12, we computed a principal-component EFA with the first sample using the SPSS software v.22. We verified all requirements related to item-communality [50], average item correlations, and item-total correlations [51]. The Kaiser–Meyer–Olkin (KMO) measure of sampling adequacy (which should ideally be ≥ 0.80) and Bartlett’s test of sphericity (which should be significant) ensured the adequacy of our sample [52]. Item retention was based on the recommendation that items with “fair” loadings and above (i.e., ≥ 0.40) and with low inter-item correlations (suggestive of low item redundancy) as indicated by the anti-image correlation matrix should be retained [53].

#### Confirmatory factor analysis

We used data from the second sample to conduct a CFA using the maximum likelihood estimation with the SPSS AMOS v.26 software. A previous study suggested that the minimum sample size to conduct a confirmatory factor analysis ranges from 3 to 20 times the number of the scale’s variables [54]. Therefore, we assumed a minimum sample of 240 participants needed to have enough statistical power based on a ratio of 20 participants per one item of the scale, which was exceeded in this sample. Parameter estimates were obtained using the maximum likelihood method and fit indices. Additionally, evidence of convergent validity was assessed in this subsample using the average variance extracted (AVE) values of ≥ 0.50 considered adequate [55] and meaning that a latent variable is able to explain more than half of the variance of its indicators on average (i.e., items converge into a uniform construct).

#### Gender invariance

To examine gender invariance of the CASRS-12 scores, we conducted multi-group CFA [56] using the second sample. Measurement invariance was assessed at the configural, metric, and scalar levels [57]. Configural invariance implies that the latent CASRS-12 variable(s) and the pattern of loadings of the latent variable(s) on indicators are similar across gender (i.e., the unconstrained latent model should fit the data well in both groups). Metric invariance implies that the magnitude of the loadings is similar across gender; this is tested by comparing two nested models consisting of a baseline model and an invariance model. Lastly, scalar invariance implies that both the item loadings and item intercepts are similar across gender and is examined using the same nested-model comparison strategy as with metric invariance [56]. Following the recommendations of Cheung and Rensvold [58] and Chen (2007) [56], we accepted ΔCFI ≤ 0.010 and ΔRMSEA ≤ 0.015 or ΔSRMR ≤ 0.010 (0.030 for factorial invariance) as evidence of invariance. We aimed to test for gender differences on latent CASRS-12 scores using an independent-samples t-test only if scalar or partial scalar invariance were established.

#### Further analyses

Composite reliability in both subsamples was assessed using McDonald’s (1970) ω, with values greater than 0.70 reflecting adequate composite reliability [59]. McDonald’s ω was selected as a measure of composite reliability because of known problems with the use of Cronbach’s α (e.g., [60]). The univariate normality of the four abuse scores was verified since the skewness and kurtosis values varied between -2 and + 2 [61]. Multivariate normality was confirmed through the calculation of the Mahalanobis distance. To assess divergent validity, we examined bivariate correlations between CASRS-12 scores and those on the additional measures included in the survey (bullying victimization, social support and eating attitudes) using the second sample. Based on Cohen (1992) [62], values ≤ 0.10 were considered weak, ~ 0.30 were considered moderate, and ~ 0.50 were considered strong correlations.

## Results

### Exploratory factor analysis (sample 1)

#### Factor analysis

Bartlett’s test of sphericity, χ^2^(66) = 10,278.12, p < 0.001, and KMO (0.841) again indicated that the CASRS-12 items had adequate common variance for factor analysis. The results of the EFA revealed four factors, which explained 87.14% of the common variance. The factor loadings are reported in Table 2.

**Table 2:** Items of the CASRS-12 in English and Factor Loadings Derived from the Exploratory Factor Analyses (EFA) with the First Sample, and Standardised Estimates of Factor Loadings from the Confirmatory Factor Analysis (CFA) in the Second Sample

Item	EFA	CFA
Factor 1: Psychological abuse		
1. My parents treat me with disrespect	0.93	0.89
2. I feel worthless because of the way my parents treat me	0.90	0.84
3. My parents blame me in others’ presence	0.79	0.78
Factor 2: Neglect		
4. My family pays attention to my wishes	0.93	0.78
5. I am allowed to decide for my wishes	0.93	0.95
6. I spend a restful life	0.92	0.76
Factor 3: Physical abuse		
7. When my parents punish me, it is not proportionate to my mistakes	0.96	0.81
8. I testify other members of my family are being beaten up	0.86	0.92
9. If I do not obey the rules of my family, I will be punished very hard	0.83	0.92
Factor 4: Sexual abuse		
10. An adult or some adults have tried to touch my private part	0.99	0.91
11. An adult of some people talk to me nastily	0.79	0.89
12. An adult made me look at or touch his/her private parts	0.56	0.90

#### Factor structure congruence and composite reliability

McDonald’s ω was adequate for the psychological (ω = 0.90), physical (ω = 0.95), and sexual abuse (ω = 0.92), as well as neglect (ω = 0.92).

### Confirmatory factor analysis (sample 2)

CFA indicated that fit of the four-factor model of the CASRS-12 obtained in the EFA was acceptable: χ^2^/df = 125.73/48 = 2.62, RMSEA = 0.063 (90% CI = 0.050, 0.077), SRMR = 0.027, CFI = 0.981, robust TLI = 0.974. The standardised estimates of factor loadings were all adequate (see Table 1). The convergent validity for this model was adequate, as AVE = 0.80.

#### Composite reliability

McDonald’s ω was adequate for the psychological (ω = 0.88), physical (ω = 0.92), and sexual abuse (ω = 0.93), as well as neglect (ω = 0.87).

### Gender invariance (sample 2)

As reported in Table 3, all indices suggested that configural, metric, and scalar invariance was supported across gender. Given these results, we computed an independent-samples t-test to examine gender differences in CASRS-12 scores. Males showed a significantly higher mean physical abuse, sexual abuse and neglect compared to women (Table 4).

**Table 3:** Measurement Invariance Across Gender in the Second Sample

Model	χ^2^	df	CFI	RMSEA	SRMR	Model Comparison	Δχ^2^	ΔCFI	ΔRMSEA	ΔSRMR	Δdf	p
Configural	211.28	96	0.972	0.055	0.033							
Metric	239.82	104	0.967	0.057	0.034	Configural vs metric	28.02	0.005	0.002	0.001	8	0.004
Scalar	263.65	116	0.964	0.056	0.036	Metric vs scalar	23.83	0.003	0.001	0.002	12	0.021

*CFI* Comparative fit index, *RMSEA* Steiger-Lind root mean square error of approximation, *SRMR* Standardised root mean square residual

**Table 4:** Comparison of abuse scores between males and females

	Males	Females	t	df	P
Psychological abuse	8.51 ± 9.77	6.90 ± 9.02	1.716	402	0.087
Physical abuse	4.61 ± 6.07	3.13 ± 5.45	2.569	402	**0.011**
Sexual abuse	2.72 ± 3.77	1.86 ± 3.40	2.398	402	**0.017**
Neglect	15.87 ± 9.40	12.95 ± 8.45	3.268	402	**0.001**

Numbers in bold indicate significant p-values

### Divergent validity (sample 2)

To assess the validity of the CASRS-12 scores, we examined bivariate correlations with all other measures included in the present study using the total sample. All CASRS-12 subscales scores were significantly and positively correlated with higher bullying victimization (except for neglect), higher eating attitudes scores (more inappropriate eating) and lower social support (Table 5).

**Table 5:** Correlations of the CASRS-12 with the other measures on the second sample

	1	2	3	4	5	6	7	8
1. Psychological abuse	1							
2. Neglect	0.12*	1						
3. Physical abuse	0.76***	0.11*	1					
4. Sexual abuse	0.74***	0.10*	0.88***	1				
5. Bullying victimization	0.59***	0.07	0.55***	0.56***	1			
6. Social support	−0.41***	−0.30***	−0.39***	−0.39***	−0.40***	1		
7. Eating attitudes	0.32***	0.15**	0.34***	0.30***	0.27***	−0.44***	1	
8. Age	−0.03	−0.03	−0.03	−0.01	−0.01	−0.05	0.04	1

*p <.05; **p <.01; ***p <.001

## Discussion

Arab countries have levels of child abuse that are among the highest globally. We believe that providing an Arabic brief measure of child abuse that permits to decrease respondent burden and costs of data collection while preserving data quality may be potentially helpful for clinicians and highly useful for researchers and policy makers in the developing Arab countries. We thus aimed through the present study to develop and validate a brief and psychometrically sound version of the CASRS, as a reliable and valid alternative to the already existing 38-item version that has been widely used in the Lebanese context. As expected, we found good model fit for the four-factor solution, adequate composite reliability, good divergent validity, as well as configural, metric, and scalar invariance across gender. One potential strength of this scale is that it assesses the four universally consensual dimensions of the child maltreatment construct (i.e., Physical abuse, Psychological abuse, Sexual abuse, Neglect) [3] regardless of whether there was injury [1, 2] through only 12 items. In light of these findings, we potentially encourage clinicians and researchers to use this scale as a valid and reliable measure of child abuse among Arabic-speaking populations.

While there have been a range of measures to assess child maltreatment, their cross-cultural validity is still largely unknown; especially in certain contexts where data is yet scarce [63]. However, many aspects of childhood trauma are largely influenced by culture. Cultural norms and values normalize to some extent some forms of abuse/neglect in some contexts. In the Lebanese society, for example, it is “normal” for children to self-care without their parents’ supervision at ages younger than what is commonly accepted [64]. Additionally, violence perpetuated within the family system can be seen to be not harmful and even adequate parental supervision in collectivist societies [64]. For these reasons, we chose to validate a brief form of a scale that was developed in a Middle East country and a collectivistic society, which might be more suitable for Arab people than all other scales that were mostly developed in Western and individualist countries.

Results revealed that both EFA and CFA yielded a four-factor structure for the CASRS-12 that mirrors the original four factors captured by the original CASRS [33], further supporting the multidimensional factor structure interpretation of the scale. In addition, the Arabic CASRS-12 showed a good internal consistency as evidenced through McDonald’s ω values ranging from 0.87 to 0.93 for the four subscales. This is consistent with the original validation study where a strong internal consistency was attested by Cronbach alpha values ranging from 0.82 to 0.95 [33], and other previous studies using the CASRS in various contexts and settings [34–36]. We consider that using McDonald’s ω strengthens our findings since it has several advantageous over Cronbach’s alpha when assessing the internal consistency of multidimensional measures [65].

In addition, our results indicate evidence for measurement invariance across gender, proving that the CASRS-12 can be applied to make valid comparisons between male and female respondents. In this vein, we found that our male participants reported having experienced significantly more physical abuse, sexual abuse and neglect compared to females. In agreement with our findings, multiple studies in different Arab countries (e.g., Palestine [66], Egypt [67], Lebanon [68]) have shown that all forms of violence and abuse are more prevalent in men than women.

In order to attest for divergent validity of the scale, and based on previous literature, we examined the correlations between child abuse dimensions and bullying victimization, eating attitudes and social support. We found that all CASRS-12 dimensions were significantly and positively correlated with more inappropriate eating attitudes and lower social support; and that psychological, physical, and sexual abuse correlated with higher bullying victimization. These findings confirm discriminant validity of the scale; and are in line with previous literature stipulating that child abuse is closely related to a range of mental health and behavioral problems including bullying victimization [12, 13], eating disorders [69, 70], and lower levels of perceived social support in adulthood [71, 72]. However, to further confirm the clinical utility of the Arabic CASRS-12, additional validation studies in clinical populations are required.

## Study limitations

Our study has certain limitations that need to be discussed. First, we used a cross-sectional design that limits conclusions about causality. Second, the study adopted an online questionnaire which, while being more effective in preserving anonymity and reducing social desirability, may be a source of limited generalizability and selection bias. Third, we used a population-based sample that precludes any conclusions about the validity of the scale in clinical samples. Fourth, further research need to investigate the stability of the CASRS-12 through test–retest reliability.

## Conclusion

Our findings support the reliability and validity of the Arabic four-factor structure CASRS-12. We hope that providing this simple and easy-to-use short form of the scale will broaden its utilization across different settings among Arabic-speaking people, and encourage cross-cultural research on child abuse, and help inform local prevention and intervention strategies.

## Data Availability

All data generated or analyzed during this study are not publicly available due the restrictions from the ethics committee. Reasonable requests can be addressed to the corresponding author.
